# Supplementary material for: Chitosan/Polyvinyl Alcohol/Tea Tree Essential Oil Composite Films for Biomedical Applications
Source: Polymers (Basel). 2021 Oct 29;13(21):3753. doi: 10.3390/polym13213753 (PMC8586949; doi:10.3390/polym13213753)
Supplement: Supplementary file 1 [file polymers-13-03753-s001.zip › polymers-supplementary-1397067.pdf]

Article

Chitosan/Polyvinyl alcohol/ Tea Tree Essential Oil composite films for Biomedical Applications

Jorge Iván Castro [1], Carlos Humberto Valencia-Llano [2], Mayra Eliana Valencia Zapata [3], Yilmar Joan Restrepo [3], José Herminsul Mina Hernandez [3], Diana Paola Navia-Porras [4], Yamid Valencia [5], Cesar Valencia [5] and Carlos David Grande-Tovar [6,*]

1 Grupo de Investigación SIMERQO, Departamento de Química, Universidad del Valle, Calle 13 No. 100-00, 76001 Cali, Colombia; jorge.castro@correounivalle.edu.co (J.I.C.);
2 Grupo Biomateriales Dentales, Escuela de Odontología, Universidad del Valle, Calle 4B No. 36-00, 76001 Cali, Colombia; carlos.humberto.valencia@correounivalle.edu.co
3 Grupo de Materiales Compuestos, Escuela de Ingeniería de Materiales, Facultad de Ingeniería, Universidad del Valle, Calle 13 No. 100-00, 760032 Santiago de Cali, Colombia; valencia.mayra@correounivalle.edu.co (M.E.V.Z.); yilmar.restrepo@correounivalle.edu.co; jose.mina@correounivalle.edu.co (J.H.M.H.).
4 Grupo de Investigación Biotecnología, Facultad de Ingeniería, Universidad de San Buenaventura Cali, Carrera 122 # 6-65, Cali 76001, Colombia
5 Área de Investigación, Desarrollo e Innovación, Disproquin S.A.S., Calle 93 Número 7u-2a, Vía Cali-Juanchito 760021, Colombia
6 Grupo de Investigación de Fotoquímica y Fotobiología, Facultad de Ciencias, Universidad del Atlántico, Carrera 30 Número 8-49, 081008 Puerto Colombia, Colombia
* Correspondence: carlosgrande@mail.uniatlantico.edu.co; Tel.: +57-53-599-484

Supplementary Materials

Table S1. Volatile compounds expressed as area percentage identified in the *Melaleuca alternifolia* essential oil

	**Compound**	**RT**	**Relative amount (%)**
Monoterpenes hydrocarbons	α-Thujene	15.96	1.1
	α-Pinene	16.35	2.7
	Sabinene	18.06	0.1
	β-Pinene	18.33	0.8
	β-Myrcene	18.72	0.8
	α-Phellandrene	19.54	0.5
	α-Terpinene	20.08	9.4
	p-Cymene	20.39	4.7
	Limonene	20.57	1.0
	1,8-cineole	20.75	3.9
	γ-Terpinene	21.91	18.4
Monoterpenes oxygenated	Terpinolene	22.93	3.7
	p-Cymenene	23.09	0.1
	Linalool	23.37	0.1
	cis-*p*-Ment-2-en-1-ol	24.50	0.2

	trans-p-Ment-2-en-1-ol	25.34	0.1
	Terpinene-4-ol	27.09	35.5
	α-Terpineol	27.41	3.5
	Trans-piperitol	27.80	0.1
Sesquiterpenes oxygenated	A mixture of compounds $C_{10}H_{16}O$+$C_{10}H_{18}O$+N.I.	29.53	0.1
	A mixture of compounds *trans*-ascaridol glicol+$C_{10}H_{14}O$	30.19	0.1
	N.I.	30.80	0.1
	$C_{10}H_{18}O_2$	31.83	0.1
Sesquiterpenes no oxygenated	α–cubebene	32.81	0.1
	Isoledene	33.82	0.1
	α–copaene	34.02	0.2
	α–gurjunene	35.26	0.6
	trans-β-Caryophellene	35.75	0.6
	$C_{15}H_{24}$	36.00	0.1
	α–Maaliene	36.29	0.1
	Aromadendrene	36.44	1.8
	Seline-5,11-diene	36.65	0.2
	Trans-muurola-3,5-diene	36.74	0.2
	α-humulene	37.01	0.2
	Aloaramadendrene	37.18	0.7
	Trans-cadine-1(6),4-diene	37.47	0.4
	γ-muurolene	37.54	0.1
	δ-selinene	37.98	0.3
	Viridiflorene	38.17	1.5
	Bicyclegermacrene	38.32	0.7
	δ-cadidine	38.95	1.6
	Mixture of compounds: *cis*-calamenene+zonarene	39.13	0.7
	Trans-cadine-1,4-diene	39.42	0.3
	epi-globulol	40.42	0.1
	A mixture of compounds: Maaliol-Palustrol	40.68	0.1
	Espatulenol	40.84	0.2
	Globulol	41.14	0.5
	Mixture of compounds:	41.41	0.4

Viridiflorol+cubeban-11-ol		
Rosifoliol	41.70	0.2
$C_{15}H_{24}0$	42.18	0.2
1-*epi*-cubenol	42.25	0.3
Cis-cadin-4-en-7-ol	42.66	0.7

Table S2. Physical-chemical analysis of the emulsions before and after adding PVA

Sample	Solids (%) *	pH*	Apparent viscosity (cP) (1/100) *	Density (g/mL)	Particle Size*		observation
					D$_{50}$ (μm)	D$_{98}$ (μm)	
CS	1.65^{b} ± 0.018	2.76^{a} ± 0.004	35^{a}	1.00	N/A	N/A	
CS/TTEO 0.5%	2.45^{a} ± 0.018	2.82^{a} ± 0.013	35^{ab} ± 0.023	1.00	0.78^{a} ± 0.004	1.56^{a} ± 0.004	
CS/TTEO 1.0%	2.10^{a} ± 0.060	2.81^{a} ± 0.004	35^{ab} ± 0.045	1.00	1.04^{a} ± 0.004	1.98^{a} ± 0.004	
CS/TTEO 1.5%	2.08^{a} ± 0.022	2.81^{a} ± 0.011	35^{b} ± 0.021	1.00	1.01^{a} ± 0.007	1.99^{a} ± 0.004	
CS	1.65^{b} ± 0.040	2.75^{a} ± 0.004	35^{a} ± 0.0032	1.00	N/A	N/A	Measurement was performed after homogenization at 3000 psi for 10 minutes.
CS/TTEO 0.5%	2.43^{a} ± 0.022	2.81^{a} ± 0.007	35^{ab} ± 0.002	1.00	0.50^{a} ± 0.007	0.98^{a} ± 0.000	
CS/TTEO 1.0%	2.07^{a} ± 0.020	2.83^{a} ± 0.016	35^{ab} ± 0.005	1.00	0.69^{a} ± 0.007	1.22^{a} ± 0.011	
CS/TTEO 1.5%	2.09^{a} ± 0.064	2.81^{a} ± 0.009	35^{b} ± 0.021	1.00	0.89^{a} ± 0.011	1.62^{a} ± 0.018	
CS	1.66^{b} ± 0.03	7.43^{a} ± 0.009	106^{a} ± 0.0012	1.00	N/A	N/A	Measurement performed after PVA addition to the CS/TTEO emulsion was added and homogenized again at 3000 psi for 10 minutes.
CS/TTEO 0.5%	2.46^{a} ± 0.04	7.44^{a} ± 0.007	74^{ab} ± 0.444	1.00	1.29^{a} ± 0.140	3.16^{a} ± 0.160	
CS/TTEO 1.0%	2.05^{a} ± 0.01	7.44^{a} ± 0.009	66^{ab} ± 0.002	1.00	1.43^{a} ± 0.031	3.44^{a} ± 0.069	
CS/TTEO 1.5%	2.10^{a} ± 0.06	4.55^{a} ± 0.013	28^{b} ± 0.444	1.00	1.60^{a} ± 0.024	3.38^{a} ± 0.049	
CS	2.74^{b} ± 0.056	2.92^{a} ± 0.011	42^{a} ± 0.003	1.00	N/A	N/A	Measurement performed two years later without homogenization
CS/TTEO 0.5%	4.00^{a} ± 0.064	2.95^{a} ± 0.004	43^{ab} ± 0.009	1.00	0.45^{a} ± 0.024	2.37^{a} ± 0.020	
CS/TTEO 1.0%	3.95^{a} ± 0.040	3.01^{a} ± 0.004	40^{ab} ± 0.012	1.00	0.32^{a} ± 0.018	0.82^{a} ± 0.018	
CS/TTEO 1.5%	3.90^{a} ± 0.013	3.15^{a} ± 0.011	42^{b} ± 0.003	1.00	0.52^{a} ± 0.004	1.42^{a} ± 0.018	

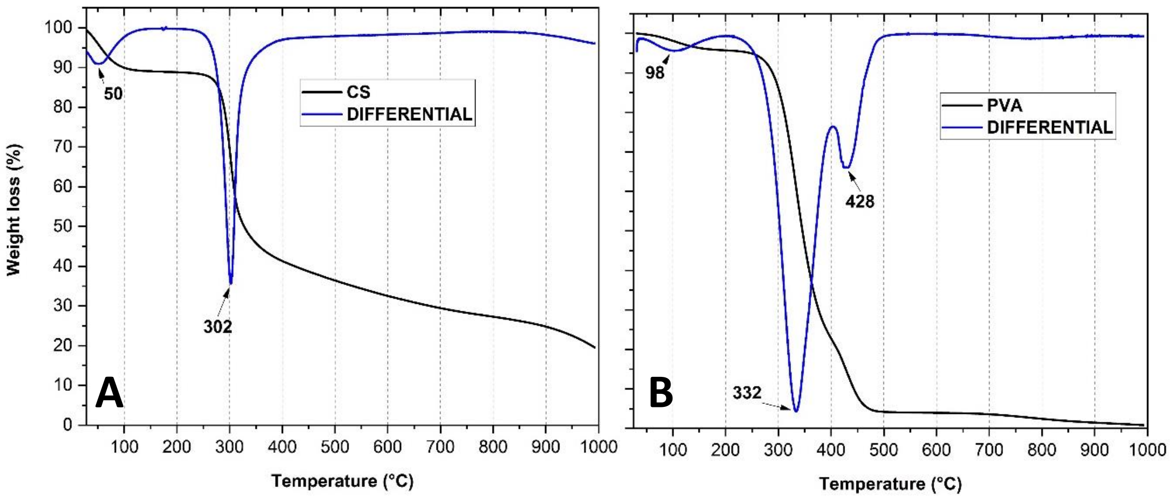

Figure S1. TGA and differential curves for CS and PVA
